# ‘All things are in flux’: China in global science

**DOI:** 10.1007/s10734-021-00712-9

**Published:** 2021-05-29

**Authors:** Simon Marginson

**Affiliations:** 1 Department of Education, University of Oxford, 15 Norham Gardens, Oxford, Oxfordshire OX2 6PY UK; 2 National Research University Higher School of Economics, Moscow, Russian Federation

**Keywords:** Research, Science, Globalisation, Higher education, International comparisons, China

## Abstract

Since 1990, a large and dynamic global science system has evolved, based on grass roots collaboration, and resting on the resources, infrastructure and personnel housed by national science systems. Euro-American science systems have become intensively networked in a global duopoly; and many other countries have built national science systems, including a group of large- and middle-sized countries that follow semi-autonomous trajectories based on state investment, intensive national network building, and international engagement, without integrating tightly into the global duopoly. The dual global/national approach pursued by these systems, including China, South Korea, Iran and India, is not always fully understood in papers on science. Nevertheless, China is now the number two science country in the world, the largest producer of papers and number one in parts of STEM physical sciences. The paper investigates the remarkable evolution of China’s science funding, output, discipline balance, internationalisation strategy and national and global networking. China has combined global activity and the local/national building of science in positive sum manner, on the ground of the nationally nested science system. The paper also discusses limits of the achievement, noting that while China-US relations have been instrumental in building science, a partial decoupling is occurring and the future is unclear.

## Introduction

For the first four decades after World War II, science and technology were largely sustained by a small number of countries in North America and Western Europe, plus Soviet Russia and later, Japan. After the advent of the Internet in 1990, instantaneous science networking developed (Wagner et al., 2015); scientists in the Euro-American duopoly became more intensively engaged (Frenken et al., 2009); other high-income countries, many middle-income countries and some low-income countries formed self-reproducing science systems; and in a process of exceptional dynamism, conditioned by almost 40 years of high economic growth, China built the largest science system in the world. China’s scientists have the second largest aggregated citation recognition after those of the United States (US), and in a small number of areas China’s scientists and research organisations are world leader. The rise of China’s science coincides with the growing R&D impact of East Asia, where both investment and the output of papers exceeds those in each of North America and Europe, and the evolution of science on a partly autonomous basis in several middle-income countries, including China, that have exhibited a pronounced growth in both national investment and national collaboration as measured by co-authored papers.

China’s rise in science, from almost zero capacity at the end of the Cultural Revolution in the mid-1970s, is the most rapid in history. Perhaps, the closest temporal equivalent is the compressed growth of science in Japan between the 1960s and 1990s but that was on the basis of earlier foundations in the nineteenth century Meiji era and on a lesser scale. The great flourishing of scientific investigation in China has exploded the belief still widely held in the Euro-American zone that Judeo-Christian civilization or Western political democracy are essential to the highest levels of intellectual achievement (Perry, 2020). Although Euro-American science and technology are part of the explanation for science in China, they are by no means the whole of it. Endogenous political–cultural factors are also at work.

Globally distributed capacity and global relations of power in science are transforming rapidly. The Euro-American countries find themselves in a world with more than one kind of modernisation, while China looks to a future in which it no longer chases Western science and technology from always behind. This brings to mind the aphorism from Heraclitus, one of the founders of Ancient Greek thought: ‘All things are in flux, like a river… Everything flows’ (Hall & Ames, 1995, p. 33). Heraclitus, who shared this ontology with foundational thinkers in China (pp. 32-36), saw a world not of being but of becoming; not a world of fixed qualities but a never-finished process. For him, change was always qualitative, opening all things to diversity, heterogeneity. For, as will be argued, to understand the rise of China’s science, it is essential to grasp both its dynamism and its specificity. National science in China does not always follow Euro-American patterns of intensive networking within the Euro-American systems and modest growth of nation-only collaboration. While science with ‘Chinese characteristics’ is still emerging, these characteristics are part of the mix.

The outlines of China’s rise in science are known. However, the paper sets out to explain the achievement and limits more precisely, positioning China in relation to both global science and endogenous factors, and analysing the interface between the national science system and global science. It is less a narrative history than a history of the present, developed within the field of higher education studies, that interprets science in China using global social theory, which combines political economy and cultural factors. The interest of the paper is in knowledge itself rather than its applications to economic accumulation. Like many studies, it is concerned with the basic science associated with academic papers, mostly conducted in universities and academies, not the full sweep of R&D and science in industry. The focus is mainland China, with the separated science systems of Hong Kong SAR, Macao SAR and Taiwan understood as part of the larger region of East Asia.

The paper begins with theoretical premises. It then discusses relations between global and national science, tendencies in global science, and the potential for national agency, before summarising science in China, including the expansion of funding and outputs, the discipline balance, citation quality, global collaboration and relations with other systems including the US. It concludes with a summary of position, strategy, trajectory and limits.

The argument draws on secondary data on science, sourced from the Organisation for Economic Cooperation and Development (OECD, 2020), and the bibliometric data in Elsevier/Scopus and Clarivate Analytics/Web of Science as reworked by the US National Science Board (NSB, 2020) and the Leiden University (2020) ranking. The paper also refers to interpretive papers on scientific output and collaboration, primarily from scientometrics. A methodological constraint in that bibliometrics contains globally recognised science rather than a fuller knowledge set including papers in both global English and in other languages. Developments at national level must be partly inferred from certain patterns in the global data including the incidence of research papers with solely national authors. This is more feasible in relation to the national sciences than in much of the social sciences and the humanities, where most work in China falls outside the global literature. Hence, the paper is confined to the disciplines involved in global conversations, those grounded in the natural sciences, rather than all research—both because of the methodological constraint and because of the central focus on global science and global/national relations in science.

## Global and national science systems

‘Scientific knowledge is produced in almost every country across the globe. Scientists are organised in global epistemic communities that codify their knowledge in peer-reviewed articles published in specialist journals’ (Wuestman et al., 2019). However, ‘communities’ of scientists are active in more than one scale. Science combines the world-spanning networks through which knowledge flows, with the national and institutional structures in which scientific activities are housed and resourced. Science combines a single communicative global science system, albeit one that combines many different conversations in fields and topics, with the world’s national science systems.

### The global scale

In social theory, scale—global, national, local, regional—is both a material domain and a domain of perception and interpretation (Herod, 2008). In his *Theory of Society* Luhmann states that the decisive step towards world society was ‘the full discovery of the globe as a closed sphere of meaningful communication’ (Luhmann, 2012, Vol. 1, p. 85). This rested on the materiality of the world as a single inter-dependent planet, and the fact of world-spanning communication systems, but also constituted a new way of seeing and acting.

The term ‘global’ does not refer to the whole world and everything in it. It refers to activities and relations that constitute a planet-scale ontology and tend to the evolution of the world on an integrated basis (Conrad, 2016; Marginson, 2010). ‘Globalisation’ means processes of convergence and integration in the global scale (Held et al., 1999). These processes are always emerging, provisional and partial. Global factors do not necessarily determine the national, or dominate local actors. Nor are global forces independent of human agents (Conrad, 2016, p. 158).  The global scale is an assemblage of structure and agency within which people and organisations act but so are nation-states and regions, the linkages of kin and locality, and organisations themselves.

This paper works in the framework of the ‘glonacal heuristic’ in higher education studies (Marginson & Rhoades, 2002). Universities and science and their individual agents are active in any and all of the global, national and local scales and also coordinate activity between them. As well as the three primary glonacal scales there are cities, sub-national regions, and the pan-national regional scale, as in the European Union (Robertson et al., 2016). Other researchers of science use scales as a tool. Leydesdorff and Wagner (2008) remark that scientists are active in ‘local, regional, national and global levels of order’ (p. 323), and Wagner et al. (2015) explores tensions between global systemic and national policy drivers of scientific collaboration. In a study of the social sciences, Heilbron (2013) uses the same four categories as Leydesdorff and Wagner. Graf and Kalthaus (2018) model three kinds of networked relations, involving differing agents: relations between national governments at global level (‘macro’), between universities at the national level (’meso’), and between researchers operating at global or national level (‘micro’), noting that ‘the network structures at different levels of aggregation influence each other’ (p. 3). Wuestman et al. (2019) contrast citations in the local, sub-national regional and national scales.

Global formation has three modes. First, there are inter-dependent systems at the world level, such as climate, or the global science system discussed here, which affect the conditions in which national and local agents operate. Second, cross-border connections and relations, for example in trade, migration and messaging (Conrad, 2016, p. 9), which can move beyond merely local impacts to achieve widely institutionalised effects. Third, worldwide diffusion of ideas, models and behaviours, encouraging the synchrony of events and sensibilities, and parallel developments, in different parts of the world. Science is implicated in all three modes. Scientific publication constitutes a worldwide system of knowledge in English. In science, there is much cross-border data transfer and people mobility. Scientific knowledge and practices are highly visible and rapidly diffused, providing favourable conditions for the continuing growth of the global science system.

### The two systems in science

**Fig. 1:**
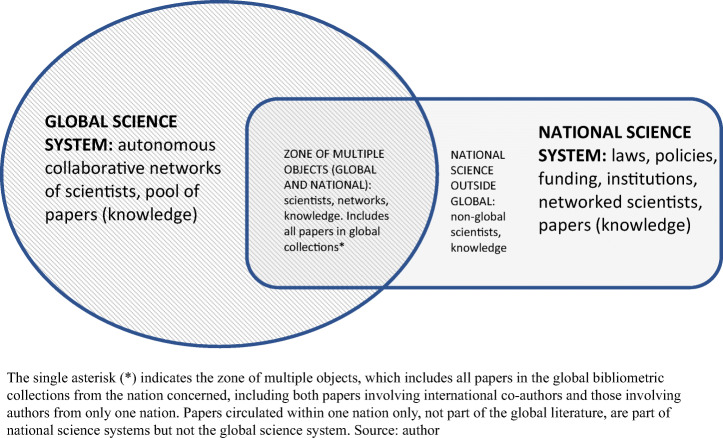
Global and national science systems and the overlap between them

By ‘system’ is meant simply a set of elements that form an interactive whole within defined boundaries. Figure 1 explains in diagrammatic form relations between the global science system and each national science system. The point to emphasise is that the two systems are heterogenous, with differing logics, though they also interact in important ways that are partly specific to each nation. This is one key to understanding the rise of science in China.

The global science system has developed only in the last 30 years. It was brought into being by global communications and it evolves in the manner of all networked information systems, expanding to include all possible connections, which explains the rapid and open character of its growth (Castells, 2000; Leydesdorff & Wagner, 2008). In science, the global system, as distinct from the national science systems, is comprised by three elements: knowledge, scientists and the professional protocols that combine scientists in cross-border relational networks and mediate the production of knowledge (King, 2011).

Codified scientific knowledge is the pool of globally published and recognised papers, nearly all of them in the English language and some with authors from more than one country. In this paper, ‘global science’ refers to only that part of science which enters into the common pool of published works. Scopus/Elsevier and Web of Science/Clarivate Analytics (Waltman, 2016), largely comprised by journal articles, define the content of this global pool. The two collections impose an inside/outside boundary in relation to globally recognised knowledge, excluding most work in languages other than English and endogenous (indigenous) thought (Connell, 2014; Marginson & Xu, 2021). The boundary line is regulated in normal scientific production in which Euro-American scientists and universities play leading roles, determining university-based notions of output and quality in science, government definitions of performance and industry understandings of useful knowledge. At the same time, published global knowledge rests on a larger combination of professional conversations, tacit understandings, unpublished data and draft papers.

Wagner et al. (2015) describe global science as ‘a dynamic system, operating orthogonally to national systems’ (p. 12). ‘Orthogonally’ means positioned at right angles. This emphasises the qualitative difference between the two systems. Whereas global science is self-managed in the accumulated interactions between scientists and has no normative centre (there is no global state), national science systems are normatively centred by nation states. National science is funded by governments, not for the production of knowledge for its own sake but for national competitive advantage, prosperity, security and survival. While global science is regulated informally by professional communities, national science is ordered formally. It consists not only of knowledge, people and scientific protocols but also laws, policies, funding, infrastructures, agencies and also institutions, which sustain important junctions between the global and national science systems. The role of universities is especially important in basic science. Although less than a fifth of R&D spending is in higher education (OECD, 2020), 85% of all science publications have at least one university-based author (Powell et al., 2017, p. 2, pp. 8-9). Though universities are mostly constituted by states, they also house global epistemic communities.

Though not all national science joins global conversation, much of it does, including work produced solely by scientists from one nation that is published in the global literature. Scientific work is often plural, with a double role, contributing simultaneously to globally networked science and to national science systems. The two systems can be understood as global/local and national/local. As shown below, in some countries, national networking and international networking have advanced simultaneously, both at a rapid rate. There is no necessary conflict between these orientations. Scientists wear two hats and many are aware of it. They can also emphasise one identity rather than another. Some are highly mobile, though most are embedded in their localities, and it is likely that for many scientists, loyalty to the discipline takes priority over country or university (Adams, 2013). Yet despite potential tensions between the two kinds of science system, there is ongoing symbiosis. Relations between national and global science systems are primarily positive sum. Each is a condition and driver of the other. ‘The sciences develop internationally, but the funding is mainly national’ (Bornmann et al., 2018, p. 931). National science and institutions provide essential conditions for autonomous global science. At the same time, the growth of global science is energised not only by the fecundity of global information but also by nations that must tap into the new science and technology continually emerging in the global science system. Access to the growing global knowledge rests on both adequate national capacity, and global engagement. Hence, most national governments expand their science budgets over time; and both governments and research universities foster the internationalisation of projects and of scientific personnel, extending national capacity while effecting global engagement. International co-authorship of papers increases continually in almost every national system as part of the burgeoning growth of networked global science.

### Autonomous national capacity

This dual global and national system structure has been associated with rapid expansion, and diversification by country of origin, of codified science in Scopus and Web of Science. Between 2000 and 2018 papers in Scopus increased by 4.94% a year, meaning that recognised knowledge was doubling every 15 years (NSB, 2020, Table S5A-2). The global diversification of capacity stands out. In the 30 years after 1987, the number of countries that contributed to 90% of bibliometric output rose from 20 to 32 (NSB, 2020, Table S5A-2). In 2018, there were 26 larger national science systems where papers increased faster than 4.94% a year (‘larger’ means that scientists authored at least 5000 papers in 2018). In 12 of those 26 countries, national per capita income was below the world average of USD $16,635 in purchasing power parity (PPP) terms. They included India, Indonesia, Brazil, Nigeria, Pakistan, Iran, South Africa and China where income was just below the mean (NSB, 2020, Table S5A-2; World Bank, 2020).

The post-1990 period has also seen a massive expansion of collaboration in science, beyond the bounds of single institutions, as indicated by co-authored papers. Collaboration entails both global networking and linkages within single national systems. The proportion of papers entailing international collaboration increased from 1.9% in 1970 prior to the Internet, to 12.4% in 1996 and 22.5% in 2018 (Olechnicka et al., 2019, p. 78; NSB, 2020, Table S5A-32), indicating intensified networking in the global system. The proportion of papers with authors from two or more institutions in one nation was much larger than the proportion entailing multiple nations and expanded by almost as much, from 35.1% in 1996 to 44.4% in 2018 (NSB, 2020, Table S5A-32). Because there are no data on papers in national languages, a full picture of national science, country by country, is not available. The trend line in nation-only collaborative papers in the global literature has to serve as a proxy for autonomous national system building.

However, the patterns of collaboration are not uniform across the world. In a review of international co-authorship since 1970, in 48 leading science countries in paper volume, Olechnicka et al. (2019) report that in 35 countries there was a marked and similar increase in the international proportion of papers. In each case, the shape of the curves was much the same: the proportion of internationally coordinated papers rose in line with the world tendency. Many of these countries had mature national science systems in which global connects multiplied continuously. Others were emerging systems where international links were substituted for national infrastructure (Chinchilla-Rodriguez et al., 2019, p. 3) and scientists engaged in international collaboration were more likely to be positioned as followers (Olechnicka et al., 2019, p. 103). Chinchilla-Rodriguez et al. (2019) cast doubt on assumptions that international co-authorship, or citation, are universal signifiers of authority. Smaller countries like Azerbaijan, Peru and Panama ‘depend almost exclusively on international collaboration for their output, with low degrees of domestic collaboration and sole authorship’ (p. 5). This indicates insufficient capacity.

The exceptional 13 cases identified by Olechnicka et al. (2019) are China, South Korea, Taiwan and Thailand in East and Southeast Asia; Russia, Poland and Romania in Eastern Europe; and Pakistan, India, Iran, Turkey, Tunisia and Brazil (pp. 80-83). Each of these 13 science systems, largely located in medium-sized or large countries, were built mostly after 1990, albeit rebuilt in Russia after the collapse of the Soviet system. In all these emerging systems, except Taiwan, the rate of growth of total papers exceeded the world average rate of growth after 2000; and in all cases national collaborations grew vigorously as the national science system developed. This modified the upward trajectory of the internationalisation curve. While in all these systems internationally co-authored papers increased markedly, as they did almost everywhere else, nationally co-authored papers also increased markedly (though again, Taiwan was an exception to the pattern). For example, in all countries in the upper part of Table 1, which includes 10 of the 13 systems, nation-only co-authored papers grew by at least 50% between 2006 and 2018 (NSB, 2020, Table S5A-32).

**Table 1:** Larger national science systems exhibiting relatively robust growth since 1970 in nation only co-authorship of papers, national and international collaborations: 2006 and 2018

Science system	Total papers 2006	Total papers 2018	Multiplier all papers 2018 compared to 2006 (2006 = 1.00)	Multiplier internationally co-authored papers (2006 = 1.00)	Multiplier nationally co-authored papers (2006 = 1.00)
Iran	11,187	54,499	4.87	6.10	5.38
Russia	36,289	93,766	2.58	1.84	5.14
Pakistan	3,302	18,609	5.64	10.57	5.10
India	42,953	150,013	3.49	3.34	3.73
China	203,829	584,407	2.87	4.93	3.57
Thailand	5,779	16,448	2.85	2.58	3.19
Brazil	32,819	73,073	2.23	3.03	2.01
South Korea	42,581	78,744	1.85	2.14	1.91
Turkey	21,650	39,449	1.82	2.83	1.58
Poland	26,457	44,997	1.70	2.00	1.55
Germany	111,975	154,301	1.38	1.76	1.08
United States	448,696	548,847	1.22	1.91	1.05
UK	116,523	161,910	1.39	2.11	0.96
Japan	125,860	119,942	0.95	1.41	0.91
WORLD	1,574,326	2,555,959	1.62	2.20	1.66

**Fig. 2:**
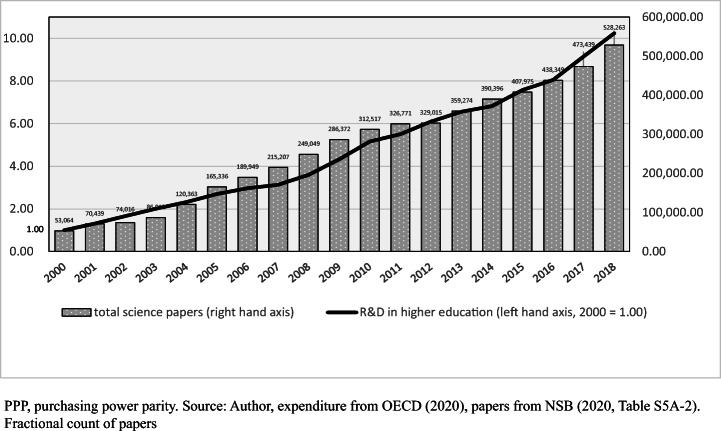
Investment in R&D in higher education in China (left-hand axis, year 2000 = 1.00) and number of science papers published by authors from China (right-hand axis): 2000 to 2018

Systems exhibiting robust growth since 1970 of national networking relative to international networking identified by Olechnicka et al. (2019, pp. 80–83) using Web of Science data. Paper totals are based on whole counts (all papers authored by scientists from the system regardless of author weightings) not fractional counts as in Fig. 2 below. Source: Author, using data from NSB, 2020, Table S5A–32

Table 1 indicates the growth in all papers, internationally co-authored papers, and papers co-authored by solely national authors, between 2006 and 2018. Data for the world as a whole, and the large established systems of US, Germany, UK, and Japan, are included for contrast. Though in most of the emerging systems internationally co-authored papers grew even faster than nationally co-authored papers, in Russia, India and Thailand the multiplier of national networking exceeded that for international network growth.

This group of countries shows itself as distinctive in various papers on science. Chinchilla-Rodriguez et al. (2019) note that China, Iran and Brazil have large systems, lower overall rates of international collaboration than most other systems, strong national networks and regional or global leadership roles (p. 6). In their study of high energy physics, Jang and Ko (2019) also identify China and Iran as countries following an ‘independent’ trajectory, with emphasis on national system building, though the growing role of collaborative infrastructure meant that all national systems became more internationalised after 2010. Similarly, Choi (2012) focuses on the ‘new rising stars’ Turkey and Korea, whose shared trajectory, underpinned by strong government investment in R&D, was national system building followed by accelerated international collaboration and global degree centrality (pp. 35-39). Choi concludes that in global science, ‘dependence’ on the ‘core actors’, meaning the Euro-American systems, is ‘slowly declining’ and predicts ‘new clusters of the global knowledge network and, as a result, more diverse knowledge (p. 39).

In developing capacity, China and the other countries in the upper part of Table 1 have pursued a dual strategy, simultaneously working both kinds of science system. They have been effectively globally engaged but only partly dependent on the global system. Building national infrastructure has enabled them to strategically select and optimise global engagement. The dual strategy is underpinned by national funding. Chinchilla-Rodriguez et al. (2019) note that countries with higher investments in R&D ‘are more scientifically independent’, have a higher ratio of national to international collaboration (p. 1, p. 6), have scientists more likely to take the lead author role in collaborative papers (p. 6), and are more likely to garner higher citations when their scientists are lead authors.

Simply put, the greater the scientific capacity of a country, the more internalised the production … the more a country invests in R&D, the greater its capacity for creating infrastructure, training skilled researchers, attracting talent and creating cohesion among domestic institutions (Chinchilla-Rodriguez et al., 2019, p. 6).

While resembling other emerging systems that have vigorously built autonomous capacity, China’s trajectory in science also shares a not quite universal East Asian dynamic. In Table 1 South Korea parallels China’s indicators at a more modest level. South Korea, Japan, Taiwan, and to a degree Singapore, share roots in Chinese civilisation (Holcombe, 2011), including comprehensive and focused states with a long view, and a Confucian ethic of self-improvement practised among both individuals and organisations (Marginson, 2013). At different times, all have sustained an advanced investment in science, and all have followed ‘catchup’ strategies designed to adapt selective features of Western (especially US American) modernisation in higher education and science. All have emphasised internationalisation and built a high volume of collaborations in US science, directing internationalisation inward to foster national capacity. All have focused on forming ‘World-Class Universities’ (Salmi, 2009) and all emphasise research in the STEM (Science, Technology, Engineering and Mathematics) disciplines. Of the regional systems touched closely by Chinese civilisation, only Vietnam and Mongolia have not followed this path.

Science in Hong Kong SAR owes more to British colonial influences than to modern China, but is also underpinned by Confucian culture and may converge more closely with the mainland in future. Universities in Hong Kong SAR and Macau SAR share the exceptional research strength of their mainland compatriots in mathematics (see Fig. 7 below).

The paper now considers the material inputs and outputs of science in China.

## Resources and outputs

Between 1995 and 2018, in the OECD countries for which data are available, investment in R&D as a share of GDP rose in 27 countries and declined in five. R&D in higher education, where the great majority of basic science is concentrated, rose almost everywhere in constant price terms. It more than doubled in the US and Canada and almost doubled in UK and Germany (OECD, 2020). In 2000, East, Southeast and South Asia comprised 25.3% of world investment in R&D. By 2017 that proportion had risen to 41.7%, mostly in East Asia, while the US share fell from 37.2 to 25.5% (NSB, 2020, Figure 14).

Between 2000 and 2018 the total funding of R&D in China multiplied by ten times. In 2018, R&D investment in higher education in China was $41.07 billion in current prices, second to the US at $74.22 billion. Japan at $19.80 billion was fourth national in the world and South Korea at $8.10 billion was the eighth largest. As in many countries, the funding of basic science in China has been proportional to outputs. Between 2000 and 2018, the funding of research in higher education in China multiplied by 10.24 in constant prices (OECD, 2020), while total papers in Scopus from China multiplied by 9.96. In 2018, China’s scientists published 528,263 papers in fractional count terms (i.e. weighted for share of authorship), having passed the total output of US scientists in 2016 (NSB, 2020, Table S5A-2). Between 2000 and 2018, China’s share of global science rose from 5.0 to 20.7% (NSB, 2020, Figure 5A-3). Xie and Freeman (2018) note that 35% of all 2016 papers in Scopus papers have at least one Chinese name or address, whether the authors were inside or outside China, and when science journals published in China in Chinese language are added to the total the proportion of all papers with Chinese authors rises to 45%.

### Outputs in specific disciplines

**Fig. 3:**
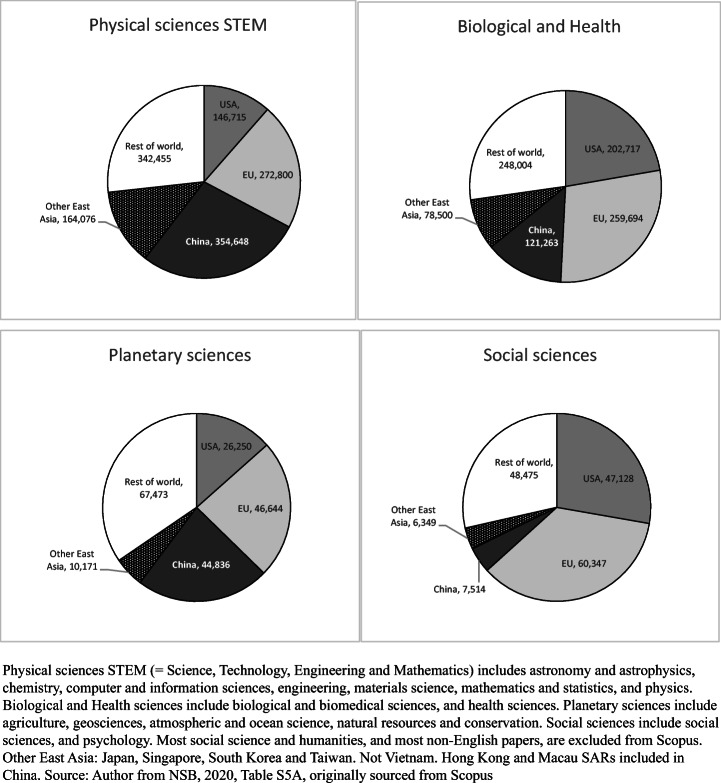
Shares of worldwide papers in Scopus in (1) physical sciences STEM, (2) biological and health sciences, (3) planetary sciences, (4) social sciences and psychology, USA, EU, China, other East Asia: 2018

Figure 3 summarises the role of China in global science in terms of 2018 papers listed by the US National Science Board, which uses Scopus data. Two factors are apparent. First, on a discipline basis, there is not one global science system but several and the weight of China varied markedly between them in 2018. China was much the largest producer in physical sciences STEM, having grown its world share of Scopus papers in this cluster from 8.5 to 27.7% between 2000 and 2018, much more than each of the US and Europe. The combined East Asia share of physical sciences STEM papers was more than 40%. China’s share of world science in biological, biomedical and health disciplines grew from 2.3 to 13.3% over the 18-year period. The priority given to biomedical sciences, health-related research and adjunct life science was higher in the US, where these disciplines constituted 48.0% of all 2018 US papers in Scopus, and in the EU (39.1%), than in China (23.0%) (NSB, 2020, Figure 5A-4). China had 22.9% of global papers in planetary science, but was a minor presence in Scopus social sciences. Because many papers in social science and psychology are in national languages, and English language papers are often focused on local or nation-bound English-speaking country issues, this cluster cannot be said to constitute a global field as such. China had just 613 papers in Scopus in social science in 2000, 1.1% of the total, rising to 4.4% in 2018 (NSB, 2020, Table S5-A). The extensive social science publishing within China in Chinese falls outside these figures. Liu et al. (2015) examine China’s publications in the Web of Science Social Science Citation Index (SSCI), for 1978–2013. The number of globally-published social science papers from Hong Kong SAR, where universities have a broad-based discipline profile similar to that in Anglo-American research universities, was almost twice that of Beijing region (p. 559).

**Fig. 4:**
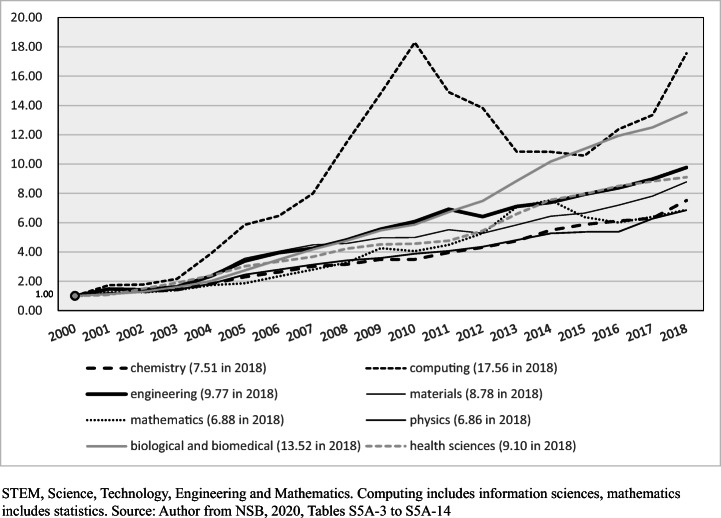
Output of published science papers in China, growth trajectory in selected STEM fields, and biological and health sciences (year 2000 = 1.00): 2000 to 2018

The skew in favour of physical sciences STEM shows itself especially in applied sciences related to construction, communications, energy, transport and other facets of national development, including engineering and computer science. Between 2000 and 2018, when compared to other STEM disciplines, there was the most rapid growth of papers in computer science in China (multiple of 17.57), engineering (9.77), materials science (8.78); and chemistry (7.51) (see Fig. 4) (NSB, 2020, Table S5A-2). China had a long global lead in 2018 in volume of papers in chemistry, materials and engineering and was first also in computer science and more narrowly, physics. Papers in engineering rose from 13,777 in 2000 to 134,542 in 2018, in chemistry from 6762 to 50,753 and in computing and information sciences from 3981 to 69,932 (see Fig. 4 for the erratic growth of computing). Meanwhile, the US share of physical sciences STEM papers fell from 23.7 to 11.5% in 2000–2018.

Interestingly, between 2000 and 2018 there was very rapid growth in biological sciences and biomedicine papers in China (13.52) and in papers in health sciences (9.10) (NSB, 2020, Tables S5A3 to S5A-14). In a study of work ‘close to the edge of the scientific frontier’ in biomedicine, as measured by the extent to which paper authors use recently published findings, Packalen (2019) notes the US and South Korea had ‘the highest tendencies for novel science’ (p. 787). Nevertheless, China was one of the world leaders in novel ideas in basic science in biomedicine, though it lagged in clinical medicine (p. 787).

### Citation recognition

The use of comparative citations as proxies for paper quality is fraught. Tahamtan and Bornmann (2019) review 41 studies of citations. The number or proportion of citations are treated as measures of value, yet citations are ambiguous. Do they express cognitive debt, or dependence, or collaboration and mutual support, or field identity, or author status?

**Fig. 5:**
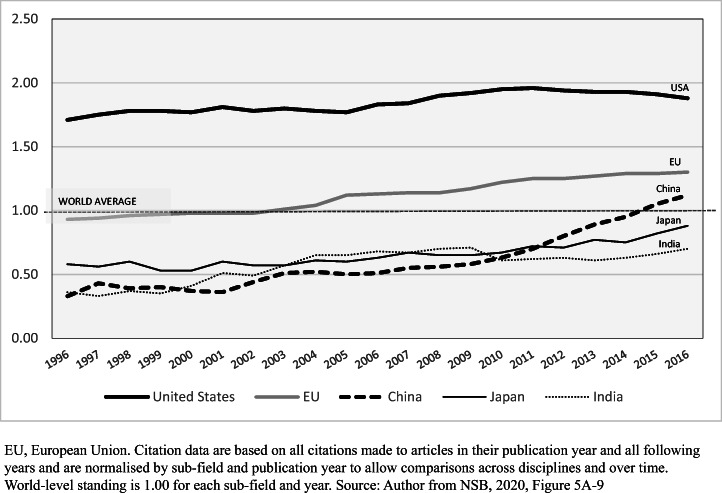
Proportion (%) of all published papers in the world top 1% by citation rate, US, EU, China, Japan: 1996–2016 (world average = 1.00, indicated by broken line)

**Fig. 6:**
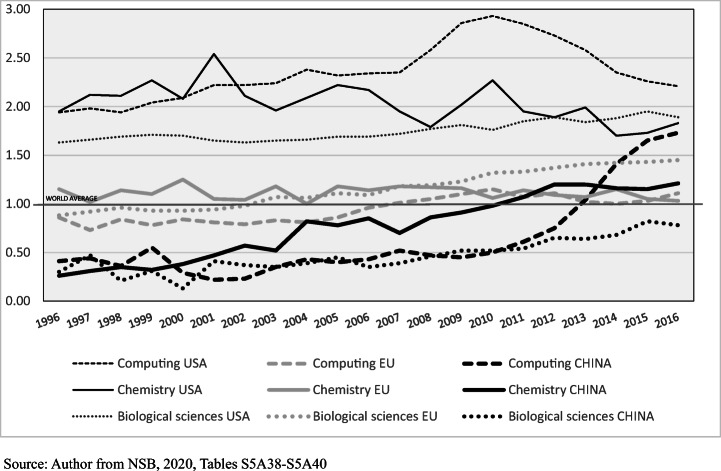
Proportion of papers (%) in the top 1% of the field by citation rate, US, EU, China. computer sciences, chemistry, biological sciences (world average = 1.00)**:** 1996 to 2016

**Fig. 7:**
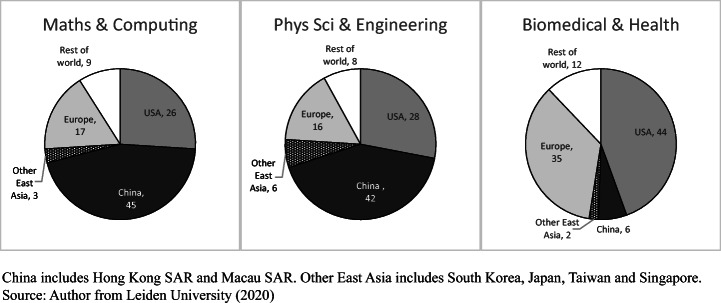
Distribution of world’s 100 leading universities in number of papers in top 5% of their field by citation, in (1) mathematics and complex computing, (2) physical sciences and engineering, (3) biomedical and health sciences, world: papers published in 2015–18

‘Our review of the empirical studies demonstrates that a paper may be cited for very different scientific and non-scientific reasons’ (p. 1635; Patelli et al., 2017, p. 1230). Different kinds of citation may share the same paper. Citations cannot provide a single standardised ‘currency’ for valuing knowledge or its creators. Nevertheless, citation data matter. Other than co-authorship, keyword studies of content similarities and differences, and data on web-usage, they are the only large-scale measures of connection and potential influence. If they do not measure value, they do measure recognition. The most commonly used citation data are average citation rates and the incidence of high citation papers. The latter are used here, in Figs. 5, 6 and 7 and Tables 2, 3, and 4.

**Table 2:** Proportion (%) of all papers that were in the world top 1% in their discipline on the basis of citation rate, by discipline, China: 2000 to 2016 (world average = 1.00)

Sciences	2000	2001	2002	2003	2004	2005	2006	2007	2008	2009	2010	2011	2012	2013	2014	2015	2016
Agriculture	0.71	0.80	0.56	0.62	0.83	0.33	0.67	0.55	0.87	0.94	0.88	1.03	0.83	0.92	0.90	0.86	1.12
biol/biomed	0.13	0.41	0.37	0.35	0.39	0.45	0.35	0.39	0.46	0.52	0.52	0.54	0.65	0.64	0.68	0.82	0.78
Chemistry	0.38	0.47	0.57	0.52	0.82	0.78	0.85	0.70	0.86	0.91	0.98	1.07	1.20	1.20	1.16	1.15	1.21
Computing	0.29	0.22	0.23	0.35	0.43	0.40	0.43	0.52	0.47	0.45	0.50	0.61	0.75	1.04	1.41	1.65	1.73
Engineering	0.28	0.28	0.38	0.49	0.41	0.38	0.40	0.38	0.46	0.51	0.60	0.64	0.76	0.93	0.94	1.09	1.17
Planetary	1.04	0.72	1.04	1.23	0.91	1.10	0.95	1.31	1.12	1.30	1.11	1.11	1.26	1.16	1.28	1.21	1.16
Health sci	0.28	0.27	0.29	0.30	0.30	0.29	0.21	0.33	0.36	0.38	0.43	0.48	0.51	0.57	0.57	0.56	0.64
Materials	0.48	0.21	0.54	0.53	0.41	0.37	0.52	0.62	0.66	0.57	0.62	0.67	0.81	0.82	0.89	1.04	1.16
Mathematics	0.91	0.89	0.62	1.04	1.02	1.17	0.87	1.23	0.79	0.93	1.02	1.12	1.12	0.97	1.03	1.44	1.63
Nat resources	0.83	0.49	0.41	0.58	0.77	0.86	0.77	1.06	0.74	0.84	0.84	0.64	0.82	0.80	0.80	1.01	0.92
Physics	0.35	0.35	0.52	0.63	0.71	0.73	0.79	0.73	0.72	0.71	0.76	0.86	0.92	0.95	0.99	1.12	1.18
All disciplines	0.37	0.36	0.44	0.51	0.52	0.50	0.51	0.55	0.56	0.58	0.63	0.70	0.80	0.89	0.95	1.05	1.12

Includes only fields in which more than 5000 papers were published in 2016. Astronomy includes astrophysics. Biol/biomed, Biological sciences and Biomedical sciences. Planetary sciences include geoscience, atmospheric and ocean sciences. Mathematics includes statistics; Nat resources, Natural resources and conservation. All disciplines include astronomy and astrophysics, social sciences and psychology, not separately listed here. Source: Author from NSB, 2020, Tables S5A-36 to S5A-49

**Table 3:** Papers in the top 5% of their research field by citation rate, selected Asian universities: 2006–2009 to 2015–2018

University	System	Top 5% papers 2006–09	Top 5% papers 2015–18	Top 5% as share of all in 2015–18	Proportion of all papers internationally co-authored 2015–18	Annual growth top 5% papers
Universities producing 500 or more papers in top 5% of their disciplinary field in 2015–18						
Tsinghua U	China	402	1451	7.3%	36.4%	15.33%
Zhejiang U	China	335	1263	5.4%	30.9%	15.89%
Shanghai Jiao Tong U	China	314	1050	4.3%	31.1%	14.35%
National U Singapore	Singapore	513	948	7.7%	66.9%	7.06%
Peking U	China	307	910	5.3%	37.4%	12.83%
Huazhong U S&T	China	114	874	5.3%	27.5%	25.40%
Nanyang Technological U	Singapore	275	861	8.6%	68.4%	13.52%
Harbin IT	China	180	790	5.5%	27.3%	17.86%
Sun Yat-sen U	China	145	742	5.0%	30.1%	19.89%
Xi’an Jiaotong U	China	111	735	4.8%	28.8%	23.37%
U Science and Technology	China	246	696	6.6%	32.5%	12.25%
Fudan U	China	230	663	4.3%	30.4%	12.48%
Central South U	China	85	652	4.7%	25.3%	25.40%
U Tokyo	Japan	656	637	4.4%	39.6%	- 0.33%
Sichuan U	China	132	624	3.8%	22.0%	18.84%
South China U Technology	China	84	624	6.4%	24.2%	24.96%
Wuhan U	China	114	622	5.1%	27.1%	20.75%
Tianjin U	China	102	607	5.1%	25.5%	21.92%
Nanjing U	China	195	595	5.1%	33.3%	13.20%
Hunan U	China	71	572	9.3%	29.1%	26.09%
U Chinese Academy of Sci	China	6	568	5.6%	24.3%	65.80%
Seoul National U	South Korea	343	543	3.5%	30.3%	5.24%
Shandong U	China	125	537	3.7%	24.6%	17.58%
Beihang U	China	40	531	5.6%	30.3%	33.29%
Tongji U	China	58	512	4.8%	31.8%	27.38%
Dalian U Technology	China	143	501	5.3%	27.8%	14.95%
East Asian universities leading their systems with less than 500 top 5% papers in 2015–18						
U Hong Kong	Hong Kong SAR	308	465	6.3%	45.9%	4.68%
National Taiwan U	Taiwan	276	303	3.4%	38.1%	1.04%
SELECTED COMPARATORS FROM OUTSIDE East Asia and Singapore						
Harvard U	USA	3593	4282	12.7%	52.0%	1.97%
U Toronto	Canada	1250	1691	7.4%	56.5%	3.41%
MIT	USA	1226	1578	14.9%	57.0%	2.84%
U Cambridge	UK	1017	1370	10.2%	69.7%	3.37%
U Michigan	USA	1308	1473	7.9%	39.2%	1.33%
ETH Zurich	Switzerland	682	933	10.0%	70.8%	3.54%
U Sydney	Australia	451	867	6.9%	57.2%	7.53%
U Utrecht	Netherlands	562	745	7.9%	60.0%	3.18%

*Does not include Israel. All universities with 500 papers published in 2015–18 and in the top 5%of their field on the basis of citations, plus selected East Asian systems not otherwise included. International is proportion of all papers (not just top 5%) that were co-authored outside the country. Source: Author from Leiden University, 2020

**Table 4:** Leading universities by number of papers in top 5% of their field by citation rate, in (1) mathematics and complex computing, and (2) physical sciences and engineering world: papers 2015–2018

University	System	(1) Mathematics and complex computing top 5% papers 2015–18	University	System	(2) Physical sciences and engineering top 5% papers 2015–18
Tsinghua U	China	300	Tsinghua U	China	830
Harbin IT	China	252	Massachusetts IT	USA	687
U Electronic S&T	China	217	Zhejiang U	China	569
Xidian U	China	201	Stanford U	USA	563
Beihang U	China	197	Nanyang TU	Singapore	533
Zhejiang U	China	197	Harvard U	USA	532
Huazhong U S&T	China	195	U California, Berkeley	USA	521
Nanyang TU	Singapore	181	U Science & Technol.	China	500
Massachusetts IT	USA	180	Harbin IT	China	455
Shanghai Jiao Tong U	China	153	Xi’an Jiaotong U	China	455
Stanford U	USA	151	Shanghai Jiao Tong U	China	439
Northwestern Poly. U	China	149	U Cambridge	UK	424
Southeast U	China	148	Huazhong U S&T	China	419
NU Singapore	Singapore	140	ETH Zurich	Switzerland	417
Wuhan U	China	136	NU Singapore	Singapore	413
Xi’an Jiaotong U	China	134	Peking U	China	407
South China UT	China	132	Tianjin U	China	400
Dalian UT	China	129	South China UT	China	392
Beijing IT	China	126	Hunan U	China	354
U California, Berkeley	USA	121	Imperial Col., London	UK	349

Source: Author from Leiden University (2020)

With the great growth of science, including emerging systems with relatively low citation rates, between 1996 and 2016, the leading science systems in Fig. 5 all increased the proportion of their papers that ranked in the top 1% of their field on the basis of citation. In China, this proportion rose from 0.33% of papers in 1996 to 1.12% in 2016 (NSB, 2020, Table S5A-35). China reached the world average citation rate for Scopus papers in 2015 (NSB, 2020, Table S5A-35). The citation rates of China’s papers are discounted by the exceptionally low recognition by American authors. The average citation rate from American papers to Chinese papers was 0.31 in 2014, though citation rate from all sources of papers by Chinese authors was 0.95 that year (NSB, 2018, Table 5-28).

Looking at specific disciplines, Table 2 shows a high rate of citation to China’s scientists in computer science and in mathematics, and the high-volume STEM disciplines engineering, chemistry, physics and materials science were above the global citation average. Figure 6 outlines China’s climb beyond the global average in computer science and chemistry, and the lower rise in biological and biomedical sciences. In computer science and chemistry, China has passed the EU. Though in all three disciplines the US-China gap has narrowed, it seems that only in computer science might it close altogether. In the US, the proportion of papers in the top 1% fell after 2010 (NSB, 2020, Table S5A-35), but a high proportion of American work is still concentrated at the top end (Leydesdorff et al., 2014, p. 609). China’s large STEM volumes are associated with a longer tail of work little read outside China. Lower citation papers do not necessarily signify lower cognitive quality or a lesser contribution to knowledge, as some such papers contribute primarily to national science. However, lower citations signify lesser global recognition and connectedness. On an average basis, the US continues to exceed China in these domains.

While global leadership in high recognition STEM science is shared between the US and China, using the proportion of papers that are in the top 1% as the indicator, the US looks stronger overall. China leads only in mathematics, with 1.63% of papers compared to 1.47% in the US. The US-China gap is large in materials, natural resources (including energy) and ecology, and biological, biomedical and health sciences, though China is closer to the US in computer science, engineering and chemistry (NSB, 2020, Tables S5A36-S5A-49). On the whole, China looks stronger when top STEM universities are compared than when whole system performance is compared, as is now discussed.

## World-class Universities

China’s flagship programmes for building ‘World-Class Universities’ (Salmi, 2009) have been the 985 project (1998) and the Double First-Class project (2017). The template is strongly influenced by Anglo-American norms (Hazelkorn, 2015). The data in Table 3 suggest that if the number of high citation papers in global science is seen as the indicator (which is a good example of an Anglo-American norm for judging performance), then World-Class Universities have been unequivocally achieved. Table 3 lists the leading universities in Asia on the basis of publications in 2015–18 inclusive that were in the top 5% of their field on the basis of citations. China overwhelmingly dominates the list with Singapore’s two universities also doing well. Table 3 also includes selected comparators from North America and Europe. In 2015–18, Tsinghua University was seventh in the world using this indicator, its 1451 papers exceeded only by Harvard, Stanford, MIT and Michigan in the US, Toronto in Canada and Oxford in the UK. Most of these universities, especially Harvard and Toronto, were sustained by exceptional paper volume in biomedicine and related fields (Leiden University, 2020).

The top Chinese universities were weaker than the Euro-American comparators in the proportion of papers in the top 5%. By this indicator, Hunan University was strongest in China (Leiden University, 2020). However, like their comparators, the leading Chinese institutions maintain strong concentrations of talent. Tsinghua had 55 Clarivate Analytics high citation researchers in 2020, compared to 188 at Harvard, 124 at the Chinese Academy of Sciences, 106 at Stanford, 62 at Berkeley and 53 at MIT. China’s University of Science and Technology had 30 such researchers and Peking University and Zhejiang University each had 24 (Clarivate Analytics, 2020, p. 20). But where the leading universities from China stand out is in annual rates of improvement. The 22 universities from China saw *annual* increases in high citation papers of between 12.25 and 65.80%. These are extraordinary figures. If this keeps up, the gap between the top US and Chinese universities, on this measure, will largely disappear, though the US universities have broader discipline profiles.

Figure 7, using Leiden University ranking data sourced from Web of Science, presents the leading 100 universities in each of three disciplinary clusters, again using top 5% papers in the comparison. In all published science together (not in Fig. 7), the top 100 was dominated by the US with 42 universities and the other Anglophone countries with 17. There were 21 universities from China. However, in mathematics and computer science, the pattern was different: 41 of the leading 100 universities were from mainland China, three from Hong Kong SAR and one from Macau SAR, compared to 26 universities from the US and 26 from the rest of the world. In physical sciences and engineering, there were 39 from mainland China, three from the SARs and 28 from the US (Leiden University, 2020).

However, China had only six universities in the leading 100 in biomedicine and health sciences. The highest placed Chinese university was Shanghai Jiao Tong at number 42.

Table 4 looks more closely at the world’s leading universities in the physical sciences STEM cluster. On the basis of the number of 2015–2018 papers in the top 5% in citation terms, adding the two lists shows Tsinghua is the world leader in STEM ahead of MIT. Zhejiang, Harbin IT, Shanghai Jiao Tong, Xi’an Jiaotong, South China UT and Huazhong UST also appear on both sides of Table 4, as do the two Singapore Universities, and Stanford and Berkeley in the US. China and Singapore occupy 60% of the positions in Table 4. China’s researchers in mathematics and computer science occupy the top seven places in the world. When the measure is switched to top 1% papers, Tsinghua with 45 was second to Harbin Institute (46) in mathematics and computing and was third with 155 in physical sciences and engineering, behind MIT (187) and Stanford (172). Tsinghua was second to MIT in the total of top 1% papers but was catching up (Leiden University, 2020).

## National/global synergy in China

Like the other systems in the top part of Table 1, China has used a dual process of capacity building at scale, drawing national and global evolution together on the ground of the national system. It has vigorously built networked activity both at home and abroad. China is the most successful of the nations that have followed the dual strategy.

In 2018, its scientists published 456,040 papers solely from China, including 280,881 (48.1%) with multiple institutions, national networking. In addition, 126,868 (21.7% of all papers) involved international collaborations. The number of nationally co-authored papers more than doubled that of internationally co-authored papers (NSB, 2020, Table S5A-32). Internationally networked papers multiplied by 4.93 in the 12 years from 2006 to 2018, yet nationally co-authored papers multiplied by 3.57, a very high multiplier for that indicator (Table 1). One sign of national system growth was that from 1996 to 2014 the international share of citations of China-authored papers fell from 51.6 to 37.7% (NSB, 2018, Table A5-42). This suggests that national capacity was augmented by both the production and use of national papers—though it may also partly reflect growth in the number of Chinese language journals indexed in Scopus (Horta & Shen, 2019).

Nevertheless, internationalisation has been also integral to capacity building since the science strategy began. When he became China’s primary leader in 1978, Deng Xiaoping took special responsibility for science and universities. He saw internationalisation as key to accelerated development, not as a source of borrowed science but as a guide to building China’s own capacity (Vogel, 2011, p. 209). Development-oriented policies included invitations to foreign scientists, especially from the US, and the creation of collaboration agreements; the outward mobility of Chinese doctoral students and mature researchers for training and collaboration; fostering English language publishing in global science through incentives (Xu, 2020); adaptation of system and institutional models from North America and Europe; and the systematic benchmarking of research, academic units and institutions with high quality foreign comparators (Wang et al., 2011). While China’s scientists were extensively engaged in open sharing with international partners in global disciplinary networks, their activities were also embedded in the party-state governance of universities and other scientific institutions (Yang & Li, 2014), ensuring that they fulfilled national objectives.

A parallel national-plus-global policy logic has shaped the formation of talent. Between 2000 and 2015, annual new doctoral graduates from China’s universities multiplied by 4.43 (NSB, 2020, S2-16). Meanwhile, from 2000 to 2017, inclusive there were 66,690 new Chinese doctoral graduates in science from US universities, one third of all foreign recipients of US doctoral degrees and equivalent to another two full years of doctoral graduates from China at the 2015 level (NSB, 2020, Table 2-4). Other graduates went to Japan, UK, Germany and France. Return rates were low in the 1980s and 1990s but rose when opportunities in China increased. Government programmes have brought back scientists from earlier cohorts, and fostered collaboration and joint appointments with those scientists who continue to work abroad. As Deng predicted, the diaspora has eventually become ‘an asset’ to China (Vogel, 2011, p. 322). Shorter stay strategies are also pursued. For example, the China Scholarship Council sent 15,645 funded scholars to 87 countries in 2012, mostly for periods of 3-6 months (Quan et al., 2019, p. 708).

In this manner, China’s science has developed as a national/global synergy. National system building and global engagement have underpinned each other. International linkages foster national talent and cognitive resources while strengthening the global integration and impact of China’s science. Building national capacity has been essential to lifting international networking from followership to equality and leadership. The expansion of the global science system has carried the growth and improvement of China’s science along with it; while increasingly, China’s scientists have become main contributors, moving into a more productive and formative role that constitutes a more fruitful engagement (Lee & Haupt, 2020). Like the US, China has created a high volume of global knowledge while tapping the globalisation of science for national purposes; though arguably, China has been less globally universal and central than US science, which remains the most effective system in mobilising people and ideas from across the world.

The respective roles of national networks and global networks vary by field. Quan et al. (2019) use the Web of Science collection to examine China’s international collaborations between 1980 and 2016, in three discipline clusters; medical fields, natural sciences and engineering, and social sciences and humanities. In natural sciences and engineering, where Chinese researchers are strong in papers and citations, ‘international collaborations constitute only a small minority of the total Chinese output’ except in general physics (p. 710) which is highly collaborative worldwide. Chinese authors were lead or corresponding author on 61.2% of all collaborative papers (p. 709). Internationally, co-authored papers led by scientists from countries other than China—often the US—tend to secure higher global citations than China-led papers (p. 707). China’s highest rates of international collaboration were in disciplines where Chinese researchers were rarely first named authors and the country had a modest presence, such as astronomy and economics (p. 710). This contradicts the widely held assumption in science literature of a universally positive relation between quality and international co-authorship (e.g. of many, the summary in Graf & Kalthaus, 2018; for a sceptical view see Chen et al., 2019). Arguably, this assumption is partly sustained by Euro-American centric beliefs that collaboration means collaboration into the Euro-American duopoly and for scientists from other systems this is *necessarily* a step up in quality. The positive relation between internationalisation and scientific quality applies some of the time, but not for all nations, sites, sites or disciplines all of the time.

Kwiek (2020) finds that in aggregate ‘papers involving national collaboration have a higher impact on global science than international collaborations in only five countries’. These are the US, China, France, Romania and Poland (p. 16). This indicates the potency of the large national networks in the US and China. Chinchilla-Rodriguez et al. (2018) find that while China and India both gain less citation recognition from their international publications than is the case in many other countries, global citation rates for nationally led and authored papers are higher in China (p. 1493). The higher quality of nation-only papers suggests that China’s strategy of nation building in science continually enhances itself. In Confucian self-cultivation, the template is external, the framework of social relations in which the individual is embedded, but the achievement always comes from within (Li, 2012). So it has been with the evolution of science in China.

### International position and connections

As Table 3 shows, in China’s leading research universities, the proportion of 2015–18 papers that were internationally co-authored exceeded the 21.7% for all 2018 papers in China: for example, 37.4% at Peking University. Between 2006 and 09 and 2015–18, Tsinghua’s international collaboration ratio rose from 22.8 to 36.4%. Ratios were significantly higher in universities in the US and Europe (Leiden University, 2020). Given that US scientists have many potential domestic collaborators, the facts that 39.2% of all papers in 2018 entailed international collaboration, and 52.0% of all Harvard papers from 2015 to 2018 likewise included international co-authors, indicate the exceptional level of global engagement among US scientists (NSB, 2020, Table S5A-32; Leiden University, 2020).

With whom do China’s scientists collaborate? The US National Science Board (NSB) uses an index of collaboration in which 1.00 is the expected number of co-authored papers between two countries, relative to the overall patterns of collaboration of each. When the index exceeds 1.00, collaboration is more intensive than expected. The NSB data show that the world’s major science blocs have differing patterns of networking. Scientists in European nations are intensively engaged with each other: nearly all collaboration rates between country pairs in Europe exceed 1.00, largely because of the European funding system, which encourages cross-country teams that engage many countries (Kwiek, 2020). However, the internationalisation of European science is primarily internal to Europe, not worldwide, except for strong networks into the US. The US has broad-ranging networks with the larger science countries but especially intensive connections only with neighbouring Canada and Mexico, with Israel and with China. China’s connections are also localised. In 2018, its scientists had intensive relations with regional Singapore (1.91) and Taiwan (1.68), growing links to Pakistan (1.40), Anglophone Australia (1.18) and the US (1.17). The index for Japan declined from 1.51 in 2006 to 1.01 in 2018 (NSB, 2020, Tables S5A-33 and S5A-34).

A further measure is the volume of co-authored papers. As Table 5 shows, scientists in China published over 5000 co-authored papers in 2018 with those in the US, UK, Australia, Canada, Germany, Japan, Singapore—where almost one third of all collaborative papers are with China—and France. They also co-authored more than 1000 papers in South Korea and Taiwan in East Asia; Austria, Belgium, Denmark, Finland, Italy, Netherlands, Poland, Spain, Sweden in the EU; Norway, Russia and Switzerland also in Europe; and Brazil, India, Iran, Malaysia, New Zealand, Pakistan, Saudi Arabia and South Africa in the rest of the world. The Table shows great growth in China’s co-authorships, and also that the US was much more extensively networked, especially in Europe. Only with Australia and Singapore did China’s co-authorships equal or exceed the connection of other countries in Table 5 into the US.

**Table 5:** Principal nation-to-nation instances of co-authored science papers, 5000 papers or more, China and US: 2018 and comparison with co-authored papers in 1996 (1996 = 1.00)

Country pair	Papers 2018	1996 = 1.00	Country pair	Papers 2018	1996 = 1.00
China-US	55,382	26.10	US-China	55,382	26.10
China-UK	14,763	21.74	US-UK	28,616	4.06
China-Australia	13,138	46.42	US-Germany	23,616	3.44
China-Canada	9449	18.75	US-Canada	21,968	3.29
China-Germany	8206	14.03	US-France	15,422	3.42
China-Japan	8024	9.47	US-Australia	13,939	6.03
China-Singapore	5563	46.00	US-Italy	13,804	4.31
China-France	5472	19.83	US-Japan	11,533	2.00
			US-Spain	10,236	5.91
			US-Netherlands	9984	4.64
			US-South Korea	9761	5.73
			US-Switzerland	9403	4.03
			US-Brazil	8671	7.42
			US-India	8058	5.81
			US-Sweden	7034	4.07
			US-Belgium	5171	5.03

Articles listed on a whole count basis, not a weighted basis according to proportion of authorship; that is, each nation listed in authorship of a paper counts as one. Source: Author from NSB, 2020, Table S5A-33

Yuan et al. (2018) use data from the National Natural Science Foundation of China to map joint grants between researchers from China and 75 other countries between 2006 and 2016. Collaborations with US-based scientists constituted 53.3% of cases, including 8505 individual grants. The second largest group was with collaborators in the UK (1322, 8.3% of cases), followed by Australia (6.6%), Canada (6.3%), Japan (5.0%) and Singapore (4.1%) (pp. 411-412). A growing proportion of activity involved Australia, Netherlands and Spain while a decreasing proportion was with Japan, Germany and Sweden (pp. 401-402, pp. 411-412). In total 7.4% of all of the grants involved regional collaborations with Belt and Road project partners, mostly in Singapore and South Korea (pp. 416-417).

Another means of examining global connections in science is through network analysis that maps the number of connections (‘edges’) between different nodes. Often national science systems are modelled as single nodes despite the high level of aggregation across diverse networks that is entailed. Working with Web of Science data, Zhang et al. (2018) find that ‘the centrality of the US is much higher than that of all countries studied’ (p. 1079). In relation to China, comparing 2001–2005, 2006–2010 and 2011–2015, the authors highlight ‘China’s sharp increase in prominence’ in international collaboration (p. 1075). China moved from fourteenth place in terms of unweighted centrality in 2001–2015 and tenth in 2006–2010 to seventh in 2011–2015 (Table 5). Unweighted centrality simply measures the spread of connections across countries. However, China’s ‘weighted centrality’, which takes into account the connections of those which whom its scientists collaborate, was higher than its unweighted centrality. In this ranking it moved to fifth place in 2011–2015, just below France, primarily because of China-US linkages (p. 1083). The authors remark that ‘China’s overall publication volume is second only to the US, but China’s centrality is even lower than that of the Netherlands’, over the whole 2000–2015 period (p. 1079). All of the larger East Asian systems, China, Japan and South Korea, are less ‘globally central’ than those in the English-speaking world and Europe (p. 1083). Nevertheless, this does not necessarily indicate a failure of merit or even connectivity. It indicates the global position of the emerging systems, which have developed partly outside the Euro-American duopoly, which confers on all of its members the same zone of multitudinous edges, and encourages a free-wheeling substitution of other European connections in place of domestic ones, especially in the smaller European systems. The patterns look different when the EU is rendered as a single node. The finding that China, Korea and the others are less ‘central’ is a function of the methods used to calculate network centrality.

Scientometrics often struggles to understand this. In a study of global networking in photovoltaics research between 1980 and 2015, Graf and Kalthaus (2018) identify an overall increase in degree centralisation. Over time, all high publishing countries joined the central group, including emerging China, South Korea and Taiwan (p. 7). However, the researchers also remark that European countries were more intensively networked with other systems than are the Asian countries. ‘In general, Asian countries seem to have a higher degree of internal interaction than European countries in the last period’ (p. 7). While scientists in Taiwan and South Korea ‘are very well connected nationally they are not as strongly connected internationally’ (p. 7). Taiwan, China and Korea had more nationally centralised systems than Germany, France and US (p. 8), and centralisation was inversely related to global embeddedness and by implication, the successful evolution of science. Graf and Kalthaus conclude that despite their major growth in papers the Asian systems ‘do not fully exploit their knowledge sourcing potentials’ (p. 12). This judgement reflects a misplaced expectation that Euro-American collaboration patterns are universal, notwithstanding the specific character of the multiple country partnerships of internally networked Europe, and also reflects a failure to grasp the fact of heterogenous trajectories in national science, as well as a failure to fully understand national networking and infrastructure building in the emerging systems.

Likewise, in their review and comparison of national science systems, Olechnicka et al. (2019), who use a centre-periphery theorisation that implies a single hegemonic global centre, are unable to successfully explain the rise of China. They acknowledge the growth of science in East Asia and parts of Europe that have been transformative at global level (pp. 92-94). ‘The recent examples of Singapore, South Korea and, in particular, China indicate that transfer from the periphery to the semi-periphery, or even to the core, is possible’ (p. 177). Yet elsewhere, they argue the tendency to ‘deconcentration’ is ‘slight’ (p. 16); or alternately, that China has become a ‘scientific superpower’ artificially through national investment rather than genuinely in the scientific sense through ‘networks and collaboration’ (p. 177). Again, the difficulty posed by China for conventional analysis is that its weight of scientific output does not correlate to the kind of international co-authorship patterns or measured network centrality that is exhibited by Euro-American systems conventionally judged by the proportion of articles that involve international co-authors. In specific discussion of China, Olechnicka and colleagues argue that Chinese science is constrained by cultural and organisational factors, including governmental intervention, bureaucratic systems and the Confucian tradition, which allegedly fails to value either ‘collaborative behaviour’ between scientists or or ‘critical thinking and the expression of personal opinions’ (pp. 155-156). Without evidence, they state that these impediments ‘may hinder further expansion’ of science in China (p. 177). The authors both acknowledge China’s global impact and then deny it, essentially because China does not have a Euro-American culture or trajectory and is therefore incomprehensible to them.

### China and the United States

All analyses find that US scientists are well networked almost everywhere (e.g. Choi, 2012; Wagner et al., 2015). As Olechnicka et al. (2019) state, ‘the contemporary global scientific network is woven around the US’ (p. 92). Its scientists publish more than one thousand papers with scientists from 41 other countries, compared to 30 countries in the case of scientists from China. The global network analysis by Leydesdorff et al. (2014) concludes that ‘the USA can be considered a major partner of all EU nations, and China is also well connected, albeit most frequently to the USA’ (p. 608). ‘China is integrated into the network through the USA’ (p. 612). This all suggests that the US is much the largest player in the Euro-American duopoly that has long dominated global science, that China regardless of its gathering power in science has no such global role, and that the China-US relationship has been one key to China’s evolution.

Table 5 underlines the weight of China-US co-authorship. Between 1996 and 2018, the annual number of papers involving scientists in the two countries rose from 2122 to 55,382, reaching more than twice the level of the next largest collaboration in global science, that of US-UK (NSB, 2020, Table S5A-33). In 2018, 25.7% of all US international co-publishing was with authors from China, and 43.7% of all China’s co-publishing was with authors from the US (NSB, 2020, Table 5A-2). China-US collaboration exceeded the co-authorship between China and all countries in Europe taken together. This indicates the degree to which China’s global engagement, including people mobility, research grants, and templates for benchmarking scientific units and universities, has been articulated through the relationship with science in the US. Models and practices from Japan, UK, Germany and other countries might have contributed markedly, alongside American ideas, helping to foster a useful diversity of approach in China, but have been much less important.

Packalen (2019) remarks: ‘China’s special relationship with the United States in science has helped to propel it to the scientific frontier’ (pp. 804-805). The relationship has been highly focused. Adams and Gurney (2018) find that in the 2002–2011 period, 77% of collaborations between US authors and Chinese authors involved only those nations, disrupting the patterns of collaboration elsewhere. ‘It is both expanding and less multilateral … The rapidly growing US-China axis is the most clearly and consistently bilateral’ (pp. 3-4). This indicates that the relationship has been a high priority in the US as well as China. China-US relations in science have been sustained by all of the cooperation between disciplinary researchers, university-to-university collaboration and national policies in both countries. Since 1979, the US-China Agreement on Cooperation in Science and Technology has involved 50 interagency agreements with federal US agencies and supported thousands of US-China cooperative programmes (Lee & Haupt, 2020, p. 3). This structure legitimates a larger volume of bottom-up passages, transfers and activities.

Through the building of extensive practical ties US and Chinese scientists have each become essential to the other. The relationship has also evolved. On the China side, US institutions, personnel and knowledge were long central to the strategy of modernisation and catchup. On the US side, the largest direct benefit has been the contribution of Chinese doctoral students and postdoctoral researchers to research in American universities, especially in the physical sciences STEM disciplines. Many Chinese scientists have been recruited to ongoing positions in US institutions and agencies. Of the 233,600 temporary visa holders enrolled in US higher education in 2018, 84,480 were from China (36.2%) (NSB, 2020, Table S2-14). Of the 42,717 temporary visa holders enrolled in the US at doctoral level in 2015–2017, 64.2% in engineering and 51.5% in physical sciences were primarily supported financially as research assistants (NSB, 2020, Table S2-3).

Nevertheless, it would be misleading to assume that China’s contribution to US science is fundamentally that of providing talent for assimilation into the US science system, though some in the US might see it that way. First, China’s talent is not wholly assimilated, as indicated by the willingness of some Chinese scientists in the US to be attracted back to China and others to develop joint appointments or dual careers, one in each country. Second, within collaborative projects Chinese scientists play a major part, or the major part, in cutting edge science. Lee and Haupt (2020) analyse 5 years of China-US co-authored papers, from 2014 to 2018 inclusive, in the Scopus data set. Among the 500 bilateral papers with the highest number of citations, on 49% of them the lead authors were scholars whose primary affiliation was with an institution in China, US scholars led on 28% of the papers and scholars with joint country affiliations led on 23% (p. 10). In the production of these high citation collaborative papers, Chinese government funding played a larger role than did US government funding. Over the period 2014 to 2018, seven Chinese funding agencies supported 114,268 publications while three US agencies supported 32,730. The most important single agency, with projects that generated 74,827 publications, was the National Natural Science Foundation of China (p. 11). The authors emphasise that in the process of engagement of US researchers into Chinese science, supported by China’s funding, there has been a transfer of China’s cognitive achievements back into the US. Whereas the China-US relation was long understood in the US as a process of US science developing science in China, Lee and Haupt’s (2020) data indicate a relationship of equals.

academic freedom, as a fundamental US higher education value, has allowed US researchers to partner, collaborate, and extend their scholarship beyond national borders as independent, “bottom-up” actors. This study demonstrates the successes of US research collaborations with China and the ways that the nation-state benefits. This study also challenges the overly simplified political rhetoric that China is dependent on or a threat to US scientific research. Our findings suggest the reverse: China is a major player in US-China research collaboration, via growth, via funding, and via intellectual leadership (Lee & Haupt, 2020, pp. 14-15).

Notwithstanding the nesting of China’s science in the state, always closer than in the US, China’s scientists have also had a high level of freedom to pursue international linkages. Within the dual global/national strategy, such linkages are seen as potentially beneficial.

Since the mid-2010s, there has been growing geo-political tension between the US and China. There has been widespread diffusion within the US of the perception that China is a growing threat to American global primacy (Bush & Hass, 2019), and the contest is partly about technological leadership. Basic science is seen as one key to technological leadership. The Chair of the US National Science Board, Diane Souvaine, stated on release of the 2020 data set that: ‘Our latest report shows the continued spread of S&E [science and engineering] capacity across the globe, which is good for humanity because science is not a zero-sum game…However, it also means that where once the U.S. was the uncontested leader in S&E, we now are playing a less dominant role in many areas’ (Souvaine, 2020). That tendency is indisputable: the more open question is the US interpretation and response. Within the framework of geo-political competition, scientific cooperation, cross-border people mobility in science and the preparation of joint papers can be seen as embodying potential threats to national security. Lee and Haupt (2021) note the potential of ‘scientific nationalism, which essentially views scientific research as a means to primarily advance the nation-state’ to diminish ‘scientific globalism’ in the collaborative global science system.

In 2020 and early 2021, there were signs that US security concerns (Lloyd-Damnjanovic, 2018) would inhibit future collaboration, including the doctoral education of Chinese students in the US, faculty mobility, joint appointments and common projects and papers. Special American hostility was directed against the One Thousand Talent programme which brought US-based Chinese researchers back to China (US Government, 2020). There appears to be a systematic US government drive, emanating from national security and intelligence agencies, to ‘decouple’ US American science from science in China (Sharma, 2020). This is not necessarily supported within US science. However, the conditions that sustained China-US cooperation in science have altered. In that cooperation, scientific nationalism and scientific globalism were aligned so that the two dimensions of science, the national science systems and the global system, grew together. US authorities now read the global/national synergy within the US as a tension. They seem indifferent to any loss of national benefit within the US, and want to reduce the benefits to China. This poses questions about the extent to which China’s scientist can continue the fecund association with their US counterparts, and China’s potential for developing partnerships in other advanced systems, and/or the extent to which global engagement will continue to feed the evolution of China’s science.

## Conclusions

The rise of China in science rests on high and sustained investment by the nation-state over more than two decades, focused national system building, the targeted deployment of international connections, resources and standards; and at the foundations of all, the Confucian ethic of self-cultivation and continuous self-improvement, as deeply felt on an individual basis as it is on a collective basis (Marginson & Yang, 2020). There is a strong bias in research funding in favour of the physical sciences STEM disciplines where scientists from China excel, as do scientists from other systems within the Chinese civilisational zone. National and global system development has been artfully combined in China, and this is key to understanding the achievement. China’s strategy has joined national system building, including collaborative science networks and the formation of high calibre research-intensive universities, which at the peak are now almost funded at the level of the leading US public universities and at the same level as their European counterparts (Usher, 2018), with a sustained and focused pattern of global engagement, collaboration and publishing that has directly fed into national system development. There has been special emphasis on productive scientific relationships with scientists in the US, though less in Europe and Japan. The outcome has been an extraordinary fecundity in the physical sciences STEM disciplines, though less emphasis on medicine and life sciences, and a less global approach to social science where national and local topics are in play and party-state authority inhibits free-wheeling inquiry.

Horta and Shen (2019) question the value of the measured outputs. Chinese scientists receive just over half the volume of citation recognition as US scientists. China’s apparent performance is boosted by Chinese language papers in Scopus, where in 2013–2017 one quarter of papers by China-affiliated researchers were in Chinese and not part of the global literature (pp. 13-15). Compared to universities in North America and Europe the range of work in China is narrow because of the STEM bias. ‘An analysis of intramural research expenditure in Chinese universities … revealed that 85 per cent of expenditure was always allocated to STEM disciplines’ (p. 11). In some social sciences and humanities, Japan outperforms China (pp. 7-8). It can be added that the quality of work below the established doctoral universities often falls away alarmingly (Altbach, 2016). Yet, the last is true of the US system also; and all these points marginally reduce rather than negate the achievement.

This paper does not provide an account of the governance of science and universities in China (Han & Xu, 2019; Li & Yang, 2014) or explore them in terms of national culture (Marginson & Yang, 2020; Yang, 2020), large and important topics in themselves. Rather, the focus has been China’s science within global science, and the relation between the global system and national system development. However, within this framework, the question of national specificity arises. Is the evolution of China’s science ultimately another manifestation of the triumph of Western modernity and US science? Does the achievement boil down to China’s success in overcoming the tensions between Sinic and Western norms (Yang, 2014), by working within a foreign language with foreign ideas? Has China pluralised global power in science solely on Western terms? To what extent is there something new?

Those who foresee limits to the rise of science in China decry it as a Western imitation, with the hidden implication that it can never be as good (or at least as authentic) as the original; or alternately, declare it has/will hit a ‘glass ceiling’ because it is not Western enough (Altbach, 2016), for being nested in a one-party state, it is bureaucratic, endemically corrupted by power, and prone to suppression of the academic capacity in epistemic decisions. Arguably, though they seem to point in opposite directions, both these familiar critiques underestimate the specificity of the Chinese context and the power of national and local agency. The scale of the state is larger in East Asia and China’s political culture does not need a Western separation of powers to function. It works on the basis of advanced levels of devolution within continuing central control. China locates science in a system of governance and self-governance that has synthesised Leninism, the Imperial tradition and Western modernisation at a high level of performance. This is not a Western system and it carefully manages the extent that it draws on the West, for example by fine-tuning the incentives to publish in global journals. Since Deng, internationalisation has been adaptive rather than isomorphic. There is much discussion of top-down decision-making, party-state interference in decisions about projects, the frenetic performance driven management of academic work in universities, and the power of *guanxi* networks to disrupt decisions on the basis of merit (e.g. the survey in Han & Appelbaum, 2018; The Economist, 2019). There is as yet no evidence that these elements are fundamentally disabling, and China’s science continues to chalk up awesome milestones except in the restricted social sciences. It draws ongoing energy from its practitioners. As Perry (2020) notes, the party-state promotes ‘a mutually advantaged state-scholar nexus’ (p. 1), deeply attractive to the peak universities that are positioned as national and now global leaders. As long as the system is consensual and productive and works with a growing talent pool, who can forecast its limits?

China’s science embodies ‘Chinese characteristics’ in policy and organisation, including a paradoxical combination of free science abroad and political control at home, where the relation between global and national functions on the basis of separation rather than synergy. Intellectual content is another matter. Horta and Shen (2019) state that in pitching work to the global literature, researchers may neglect local issues and problems (p. 15), and adopt global theories, methods and mentalities without bringing ‘indigenous knowledge to the global knowledge pool’ (p. 16). Though there is ongoing potential for the humanities and some social science to draw on the well of Chinese scholarship, the science disciplines in China are the Euro-American disciplines, though their priorities and adaptations are more endogenous. What are the prospects of new fields of scientific knowledge emerging in China? No one knows the answer to that, but in February 2020 the government reduced the role of Web of Science indicators in academic assessment (Nature, 2020), opening the way to the development of more endogenous definitions of legitimate knowledge. This in turn makes more possible a ‘China going out’ (Xu, 2020) with new modes of thought.

The evolution of science in China has disturbed long-standing Western beliefs about global relations in science. Prior to the 1990s, world science functioned as a duopoly of North America and Europe with outliers in the European settler states, including Israel, and Japan. The US was overwhelmingly strong. A range of countries have now used the fast-expanding global science system to build capacity. Some emerging scientists become attached to the duopoly systems, especially the US, while in countries which have accumulated national capacity, scientists can either do this or choose a more autonomous path. The most successful emerging systems are those which invest strongly at national level and use their global engagement to build national capability as well as vice versa. These developments are fostering a more complex multi-centred global order. China is leading this process, which is as yet insufficiently understood in studies of science.

China’s development in science has been much advanced by collaboration with US scientists. It remains to be seen whether, if the China-US relation in science is weakened, China’s national science system will continue to grow its national and global contributions. The weakening may be less than the American authorities expect. In the early months of the Covid-19 pandemic in 2020 there was a high level of collaboration between biomedical scientists in the two countries (Lee & Haupt, 2021). It would be unwise to underestimate either the autonomous potency of unregulated global science and its capacity to expand and include, nor the capacity of the state in China to reposition, augment and evolve national science. Cooperation with European systems offers new tools for reflexivity. Meanwhile, a year into the pandemic China continues to grow the national budget for science. These open-ended potentials underline the wisdom of Heraclitus. ‘All things are in flux, like a river’. Do not expect a stable equilibrium. Global science is continually changing. The future cannot be foreseen. ‘Everything flows’.

## References

[CR1] Adams J (2013). The fourth age of research. Nature.

[CR2] Adams, J., & Gurney, K. (2018). Bilateral and multilateral coauthorship and citation impact: Patterns in UK and US international collaboration. *Frontiers in Research Metrics and Analytics, 3*(12). 10.3389/frma.2018.00012.

[CR3] Altbach, P. (2016). China’s glass ceiling and feet of clay. *University World News* 19 February.

[CR4] Bornmann L, Adams J, Leydesdorff L (2018). The negative effects of citing with a national orientation in terms of recognition: National and international citations in natural-sciences papers from Germany, the Netherlands, and the UK. Journal of Informetrics.

[CR5] Bush, R. and Hass, R. (2019). The China debate is here to stay. *Brookings*. https://www.brookings.edu/blog/order-from-chaos/2019/03/04/the-china-debate-is-here-to-stay/.

[CR6] Castells, M. (2000). *The rise of the network society. Volume I of The Information Age: Economy, Society and Culture* (2nd ed.). Oxford: Blackwell.

[CR7] Chen K, Zhang Y, Fu X (2019). International research collaboration: An emerging domain of innovation studies. Research Policy.

[CR8] Chinchilla-Rodriguez Z, Miguel S, Perianes-Rodriguez A, Sugimoto C (2018). Dependencies and autonomy in research performance: Examining nonoscience and nonotechnology in emerging countries. Scientometrics.

[CR9] Chinchilla-Rodriguez Z, Sugimoto C, Lariviere V (2019). Follow the leader: On the relationship between leadership and scholarly impact in international collaborations. PLOS ONE.

[CR10] Choi S (2012). Core-periphery, new clusters or rising stars?: International scientific collaboration among ‘advanced’ countries in the era of globalisation. Scientometrics.

[CR11] Clarivate Analytics (2020). *Highly Cited Researchers 2020*. https://clarivate.com/webofsciencegroup/wp-content/uploads/sites/2/dlm_uploads/2020/11/WS559074072-Highly-Cited-Researchers-2020_Executive-summary_Report_v5-PH.pdf.

[CR12] Connell R (2014). Using southern theory: Decolonizing social thought in theory, research and application. Planning Theory.

[CR13] Conrad, S. (2016). *What is global history?* Princeton: Princeton University Press.

[CR14] Frenken K, Hardeman S, Hoekman J (2009). Spatial scientometrics: Towards a cumulative research program. Journal of Informetrics.

[CR15] Graf, H., & Kalthaus, M. (2018). International research networks: Determinants of country embeddedness. *Research Policy*. 10.1016/j.respol.2018.04.001.

[CR16] Hall, D., & Ames, R. (1995). *Anticipating China: Thinking through the narratives of Chinese and Western culture*. State University of New York Press.

[CR17] Han, X., & Appelbaum, R. (2018). China’s science, technology, engineering, and mathematics environment: A snapshot. *PLOS One*. 10.1371/journal.pone.0195347. 10.1371/journal.pone.0195347 PMC5882148 29614123

[CR18] Han S, Xu X (2019). How far has the state ‘stepped back’: An exploratory study of the changing governance of higher education in China (1978–2018). Higher Education.

[CR19] Hazelkorn, E. (2015). *Rankings and the reshaping of higher education* (2nd ed.). Palgrave.

[CR20] Heilbron J (2013). The social sciences as an emerging global field. Current Sociology.

[CR21] Held, D., McLew, A., Goldblatt, D., & Perraton, J. (1999). *Global transformations: Politics, economics and culture*. Stanford University Press.

[CR22] Herod, A. (2008). Scale: The local and the global. In S. Hollway, S. Rice, G. Vallentine, & N. Clifford (Eds.), *Key Concepts in Geography* (2nd ed., pp. 217–235). London: Sage.

[CR23] Holcombe, C. (2011). *A history of East Asia: From the origins of civilisation to the twenty-first century*. Cambridge University Press.

[CR24] Horta, H., & Shen, W. (2019). Current and future challenges of the Chinese research system. *Journal of Higher Education Policy and Management*. 10.1080/1360080X.2019.1632162.

[CR25] Jang Y-S, Ko Y (2019). How latecomers catch up to leaders in high-energy physics as Big Science: Transition from national system to international collaboration. Scientometrics.

[CR26] King R (2011). Power and networks in worldwide knowledge coordination: The case of global science. Higher Education Policy.

[CR27] Kwiek, M. (2020). What large-scale publication and citation data tell us about international research collaboration in Europe; changing national patterns in global contexts. *Studies in Higher Education*. Published online. 10.1080/03075079.2020.1749254.

[CR28] Lee J, Haupt J (2020). Winners and losers in US-China scientific research collaborations. Higher Education.

[CR29] Lee J, Haupt J (2021). Scientific collaboration on COVID-19 amidst geopolitical tensions between the US and China. The Journal of Higher Education.

[CR30] Leiden University. (2020). *CWTS Leiden Ranking 2019*. Leiden University Centre for Science and Technology Studies https://www.leidenranking.com/ranking/2019/list.

[CR31] Leydesdorff L, Wagner C (2008). International collaboration in science and the formation of a core group. Journal of Informetrics.

[CR32] Leydesdorff L, Wagner C, Bornmann L (2014). The European Union, China, and the United States in the top-1% and top-10% layers of most-frequently cited publications: Competition and collaborations. Journal of Informetrics.

[CR33] Li, J. (2012). *Cultural foundations of learning: East and West*. Cambridge: Cambridge University Press.

[CR34] Li, M., & Yang, R. (2014). *Governance reforms in higher education: A study of China*. UNESCO https://unesdoc.unesco.org/ark:/48223/pf0000231858.

[CR35] Liu W, Hu G, Tang L, Wang Y (2015). China’s global growth in social science research: Uncovering evidence from bibliometric analyses of SSCI publications (1978-2013). Journal of Informetrics.

[CR36] Lloyd-Damnjanovic, A. (2018). *A preliminary study of PRC political influence and interference activities in American Higher Education*. Wilson Center. https://www.wilsoncenter.org/sites/default/files/prc_political_influence_full_report.pdf.

[CR37] Luhmann, N. (2012). *Theory of society, Vol. 1*. Translated by Rhodes Barrett. Stanford University Press.

[CR38] Marginson, S. (2010). Space, mobility and synchrony in the knowledge economy. In S. Marginson, P. Murphy, & M. Peters (Eds.), *Global creation: Space, mobility and synchrony in the age of the knowledge economy* (pp. 117–149). Peter Lang.

[CR39] Marginson, S. (2013). The changing geo-politics of creativity: Rise of the post-Confucian University. In M. Peters & T. Besley (Eds.), *The Creative University* (pp. 9–32). Sense Publishers.

[CR40] Marginson S, Rhoades G (2002). Beyond national states, markets, and systems of higher education: a glonacal agency heuristic. Higher Education.

[CR41] Marginson, S. and Xu, X. (2021). *Hegemony and inequality in global science: Problems of the center-periphery model*. [draft paper under review]

[CR42] Marginson, S., & Yang, L. (2020). China meets Anglo-America on the New Silk Road: A comparison of state, society, self, and higher education. In M. van der Wende, W. Kirby, N. Liu, & S. Marginson (Eds.), *China and Europe on the New Silk Road: Connecting universities across Eurasia* (pp. 255–283). Oxford University Press.

[CR43] National Science Board, NSB (2018). *Science and Engineering Indicators 2018*. https://www.nsf.gov/statistics/2018/nsb20181/.

[CR44] National Science Board, NSB (2020). *Science and Engineering Indicators 2020*. https://ncses.nsf.gov/pubs/nsb20201.

[CR45] Nature (2020). China changes tack: A new researcher-evaluation system must not reduce international collaborations. Editorial, 5 March. Nature.

[CR46] Olechnicka, A., Ploszaj, A., & Celinska-Janowicz, D. (2019). *The geography of scientific collaboration*. Routledge.

[CR47] Organization for Economic Cooperation and Development, OECD (2020). *Science and technology indicators*. https://stats.oecd.org/Index.aspx?DataSetCode=MSTI_PUB.

[CR48] Packalen M (2019). Edge factors: Scientific frontier positions of nations. Scientometrics.

[CR49] Patelli A, Cimini G, Gabrielli A (2017). The scientific influence of nations on global scientific and technological development. Journal of Informetrics.

[CR50] Perry E (2020). Educated acquiescence: How academia sustains authoritarianism in China. Theory and Society.

[CR51] Powell, J., Fernandez, F., Crist, J., Dusdal, J., Zhang, L., & Baker, D. (2017). Introduction: The worldwide triumph of the research university and globalizing science. In J. Powell, D. Baker, & F. Fernandez (Eds.), *The Century of Science: The global triumph of the research university* (pp. 1–36). Emerald Publishing.

[CR52] Quan W, Mongeon P, Sainte-Marie M, Zhao R, Lavriviere V (2019). On the development of China’s leadership in international collaborations. Scientometrics.

[CR53] Robertson, S., Olds, K., Dale, R., & Dang, Q. (Eds.). (2016). *Global regionalisms and higher education: Projects, processes, politics*. Edward Elgar.

[CR54] Salmi, J. (2009). *The challenge of establishing world-class universities*. The World Bank https://openknowledge.worldbank.org/handle/10986/2600?locale-attribute=en.

[CR55] Sharma, Y. (2020). US targets Chinese talent in drive to ‘decouple’ science. *University World News*, 12 December.

[CR56] Souvaine, D. (2020). *America’s share decreasing as global science and engineering grows*. News release, US National Science Board, 15 January. https://www.nsf.gov/nsb/news/news_summ.jsp?cntn_id=299790&org=NSB&from=news.

[CR57] Tahamtan I, Bornmann L (2019). What do citation counts measure? An updated review of studies on citations in scientific documents published between 2006 and 2018. Scientometrics.

[CR58] *The Economist* (2019). *Can China become a scientific superpower?* 12 January.

[CR59] United States (US) Government (2020). *Proclamation on the suspension of entry as nonimmigrants of certain students and researchers from the People’s Republic of China*. 29 May. https://www.whitehouse.gov/presidential-actions/proclamation-suspension-entry-nonimmigrants-certain-students-researchers-peoples-republic-china/.

[CR60] Usher A (2018). Have Chinese universities hit a plateau?. International Higher Education.

[CR61] Vogel, E. (2011). *Deng Xiaoping and the transformation of China*. The Belknap Press.

[CR62] Wagner C, Park H, Leydesdorff L (2015). The continuing growth of global cooperation networks in research: A conundrum for national governments. PLoS ONE.

[CR63] Waltman L (2016). A review of the literature on citation impact indicators. Journal of Informetrics.

[CR64] Wang, Q. H., Wang, Q., & Liu, N. (2011). Building world-class universities in China: Shanghai Jiao Tong University. In P. Altbach & J. Salmi (Eds.), *The Road to Academic Excellence: The making of world-class research universities* (pp. 33–62). World Bank.

[CR65] World Bank. (2020). *Indicators*. https://data.worldbank.org/indicator

[CR66] Wuestman, M., Hoekman, J., & Fenken, K. (2019). The geography of scientific citations. *Research Policy.*10.1016/j.respol.2019.04.004.

[CR67] Xie, Q. and Freeman, R. (2018). Bigger than you thought: China’s contribution to scientific publications. *NBER Working Paper* No. 24829. http://www.nber.org/papers/w24829.

[CR68] Xu X (2020). China goes out in a centre–periphery world: Incentivizing international publications in the humanities and social sciences. Higher Education.

[CR69] Yang R (2014). China’s strategy for the internationalization of higher education: An overview. Frontiers of Education in China.

[CR70] Yang R (2020). Political culture and higher education governance in Chinese societies: Some reflections. Frontiers of Education in China.

[CR71] Yang, R. & Li, M. (2014). *Governance reforms in higher education: A study of China*. Paris: UNESCO International Institute for Education Planning. https://unesdoc.unesco.org/ark:/48223/pf0000231858.

[CR72] Yuan L, Hao Y, Li M, Bao C, Li J, Wu D (2018). Who are the international research collaboration partners for China? A novel data perspective based on NSFC grants. Scientometrics.

[CR73] Zhang Z, Rollins J, Lipitakis E (2018). China’s emerging centrality in the contemporary international scientific collaboration network. Scientometrics.

